# Pediatric Tuina (massage) for primary monosymptomatic nocturnal enuresis: A protocol for systematic review and meta-analysis

**DOI:** 10.1097/MD.0000000000023738

**Published:** 2020-12-18

**Authors:** Muqing Liu, Yingying Li, Jin Xian, Wenlong Yang, Qing Gao, Juan Yu

**Affiliations:** a School of Acupuncture -Tuina, Shandong University of Traditional Chinese Medicine, Shandong Province; b School of Acupuncture -Tuina, Henan University of Traditional Chinese Medicine, Henan Province; c Pain Department, the First Affiliated Hospital of Shandong First Medical University, Shandong Province; d The Affiliated Hospital of Shandong University of TCM, Shandong Province, China.

**Keywords:** primary monosymptomatic nocturnal enuresis, protocol, systematic review, traditional Chinese medicine massage

## Abstract

**Background:**

Primary monosymptomatic nocturnal enuresis is one of the common diseases of preschool and school-age children and it will cause adverse effects on the healthy growth. Pediatric Tuina (massage) has been widely used in the treatment of monosymptomatic nocturnal enuresis in China. The study is conducted to summarize the current evidence on the effects and safety of Pediatric Tuina (massage) therapy for the treatment of primary monosymptomatic nocturnal enuresis in children.

**Methods:**

The following electronic databases will be searched from establishment to December, 2019: Cochrane Library, MEDLINE, EMBASE, Web of Science, World Health Organization International Clinical Trials Registry Platform (ICTRP), China National Knowledge Infrastructure (CNKI), Wan-fang database, Chinese Scientific Journal Database (VIP), Chinese Biomedical Literature Databases (CBM), and other databases, without any language restrictions. Randomized controlled trials about this theme will be retrieved. Independent reviewers will operate literature retrieval, duplication removing, screening, quality evaluation, data analyses by EndNote (X9), and Review Manager (5.3). Meta-analysis, subgroup analysis, and/or descriptive analysis will be performed based on the included data form.

**Results:**

High-quality synthesis and/or descriptive analysis of current evidence will be provided from improvement of nocturia frequency, improvement of sleep wakefulness disorder, and total efficiency.

**Conclusion:**

This review will provide evidence of whether Pediatric Tuina (massage) is an effective and safe intervention for primary monosymptomatic nocturnal enuresis in children.

**Trail registration number:**

This protocol of systematic review has been registered on PROSPERO website (No. CRD42020165107).

## 1 Introduction

### 1.1 Description of the condition

Primary monosymptomatic nocturnal enuresis (MNE), a special type of urinary incontinence, is one of the common diseases of preschool and school-age children. The Diagnostic and Statistical Manual of Mental Disorders-Fourth Edition (DSM-IV) defines enuresis as age ≥5 years old, with an average of at least 2 involuntary nocturnal leakage per week for more than 3 months, excluding congenital and acquired neurogenic dysuria.^[1]^ The international classification of diseases (ICD-10) defines that children aged 5 to 6 have at least twice a month the symptoms of involuntary leakage of urine during night sleep, and children aged 7 years and above urinate at least once a month for more than 3 consecutive months, with obvious mental and neurological abnormalities every month.^[2]^ The definition of enuresis in traditional Chinese medicine (TCM) is similar to that in western medicine, the difference point is that the age is set at 3 years old.^[3,4]^ The difference is mainly based on the following 2 reasons: firstly, the probability of using diaper was low in the past, and urine training was relatively early; secondly, the improvement of children's quality of life, the symptoms of bed wetting became more and more difficult to accept, and parents of children over 3 years old with high frequency of bed wetting also had strong treatment requirements. Therefore, Chinese medicine defines children with enuresis as the boundary of 3 years old, and excludes that the infant's body is not fully developed, the self-control ability of micturition has not yet formed, or the child is excessively playful during the day, sleeps soundly at night, and occasionally has enuresis. In this article, clinical reports of 3 years old were included in the literature.

The prevalence rate of children enuresis is 1.7% to 33.0% in many countries and regions around the world.^[5–7]^ The prevalence of enuresis in China has been 4.8% in recent 10 years, and the incidence rate is increasing.^[8]^ Clinical study showed that 64% of children with primary MNE had sleep disorders, such as snoring and teeth grinding, 42% suffered from hyperactivity, 34% suffered from inattention, and 27% suffered from timid and weak personality.^[9]^

### 1.2 Description and function of intervention

The treatment methods for primary MNE recommended by the International Children's Continence Society mainly include desmopressin and alarm bell (wake up). These therapeutic methods play important roles in the treatment of primary MNE. Desmopressin is generally used for children over 6 years old, and recurrence rate is raising when patients stop using desmopressin, or they will suffer certain side effects by using it for long time. The most common side effects in clinical studies were headache, nausea, and vomiting. When the child sleeps and enuresis at night, the alarm bell wakes the child to exhaust the remaining urine and clean the bedsheet. Through repeated training, children can feel the desire to urinate and consciously wake up to urinate. However, this scheme takes a long time to take effect and requires the cooperation and assistance of parents, with a recurrence rate of 4% to 55%.^[10]^ More effective and targeted antienuresis treatment methods are needed.

Pediatric Tuina (massage) developed rapidly and reached its peak in the Ming Dynasty, forming a unique theoretical system.^[11,13]^ As a form of TCM therapy, Pediatric Tuina is based on TCM zang-fu organ theory and meridian theory and seeks to unblock meridians, promote the circulation of qi and blood, regulate the functions and the zang-fu organs, and strengthen the body's resistance to pathogens by using various manual techniques at specified locations on the surface of the body.^[11–13]^ Studies have revealed, that delayed brain development and detrusor instability are 2 important parts of the pathogenesis of enuresis in children. Clinical articles have shown that TCM massage could regulate and improve brain function, promote brain development, transmit signals to brain and viscera through spinal nerve root reflex. Imaging examination also confirmed that manual therapy can cause signal changes in different brain reflex areas.^[14,15]^ The TCM doctors manually stimulate specific acupoints of the body which are located primarily on the fingers, palms, arms, head, and back, and include points such as shenjing (touch the child's left little finger slowly), pijing (touch the child's left thumb kindly), baihui (GV20), mingmen (GV4), shenshu (BL23), pangguangshu (BL28). Tuina manipulations include pushing, kneading, grasping, pounding, arc-pushing, and so forth. All manipulations are conducted with a light, slow, and soft touch.

### 1.3 Why this systematic review is important

Primary MNE is a common disease of children and adolescents. If not treated in time, it will cause adverse effects on the healthy growth of children. Long term nocturnal enuresis may bring greater disease burden and psychological pressure to children and their families. It causes self-esteem damage, poor school performance, mental disorders, psychological disorders, emotional disorders and many other problems.^[16,17]^ The use of pediatric tuina to treat children with primary MNE was first recorded in the early 1960s. For more than half a century, pediatric tuina has formed a systematic theory and clinical treatment system for the treatment of children with primary MNE, and achieved satisfactory results. However, the evidence was still limited based on subjectivity judgment, nonstandard measurement, and other factors. On the other hand, no related evidenced review or protocol was published. So this review is urgently needed and important.

## 2 Materials and methods

This systematic review protocol has been registered in the PROSPERO network (No. CRD42020165107). All steps of this systematic review will be performed according to the Cochrane Handbook (5.3.0).

### 2.1 Selection criteria

#### 2.1.1 Types of studies

Published Randomized controlled trial and blinded research that reported the efficacy and safety of TCM massage for primary MNE will be included. These clinical trials include experimental group and control treatment. The experimental group is the clinical literature of pediatric tuina or massage treatment to primary MNE. The control treatment. The baseline data of each group are comparable. As there is a risk of interference with the outcome, nonrandomized controlled trials will be excluded.

#### 2.1.2 Types of patients

Children aged 3 to 14 who were diagnosed as primary MNE will be included in this study.

#### 2.1.3 Types of interventions and comparisons

The types of pediatric tuina (massage) interventions should be based on meridian acupoint theory and massage therapy, including acupressure, pushing, rubbing, transiting, stroking, and chiropractics. A variety of control interventions will be included: no treatment, placebo, and other interventions (eg, acupuncture, moxibustion, drugs, physical interventions, gentle touch, and other massage therapies).

#### 2.1.4 Types of outcomes

Primary outcomes will include effective rate; reducing number of enuresis during treatment. Secondary outcomes will include the nocturnal bladder capacity; adverse effects.

### 2.2 Exclusion criteria

Exclusion criteria include:

(1) age <3 years old or >14 years old;
(2) review, case reports, animal experiment, theoretical essays, and case-control studies;
(3) massage as an adjuvant therapy and the comparative study between the treatment group and the control group with different massage regimens;
(4) clinical studies describing the wrong random sequence generation method;
(5) repeatedly published literature, only retained those with large and complete information;
(6) incomplete data and obvious errors.

### 2.3 Literature search

The following databases will be searched from inception to December 2019: Cochrane Library, MEDLINE, EMBASE, Web of Science, World Health Organization International Clinical Trials Registry Platform (ICTRP), China National Knowledge Infrastructure (CNKI), Wan-fang database, Chinese Scientific Journal Database (VIP), Chinese Biomedical Literature Databases (CBM), The search included 9 English databases (Pub Med and Embase are 2 “must be searched” databases that are recommended by Cochrane collaboration) and Chinese databases, covering the major databases for international publications and literature published in China. The following keywords use: (Massage OR anmo OR acupressure OR tuina OR manipulat∗) AND (primary monosymptomatic nocturnal enuresis OR nocturnal enuresis) AND (child∗ OR youth OR pediatric∗ OR preschool∗ OR preschool∗OR teenager∗OR juvenile OR adolescent). To search the Chinese databases, the corresponding Chinese keywords will be used. No language restrictions are formulated in our study. The search strategy is available in Table 1.

**Table 1:** MEDLINE search strategy.

#1 Title/Abstract: Massage
#2 Title/Abstract: anmo
#3 Title/Abstract: acupressure
#4 Title/Abstract: tuina
#5 Title/Abstract: manipulat∗
#6 Title/Abstract:child
#7 Title/Abstract: youth
#8 Title/Abstract: pediatric∗
#9 Title/Abstract: preschool∗
#10 Title/Abstract: pre-school∗
#11 Title/Abstract: teenager∗
#12 Title/Abstract:juvenile
#13 Title/Abstract:primary monosymptomatic nocturnal enuresis
#14 Title/Abstract:nocturnal enuresis
#15 #1 or #2 or #3 or #4 or #5
#16 #6 or #7 or #8 or #9 or #10#11 or #12 or #13
#17 #13 or #14
#18 #15 and #16 and #17

### 2.4 Data collection and analysis

#### 2.4.1 Selection of literature

**Figure 1:**
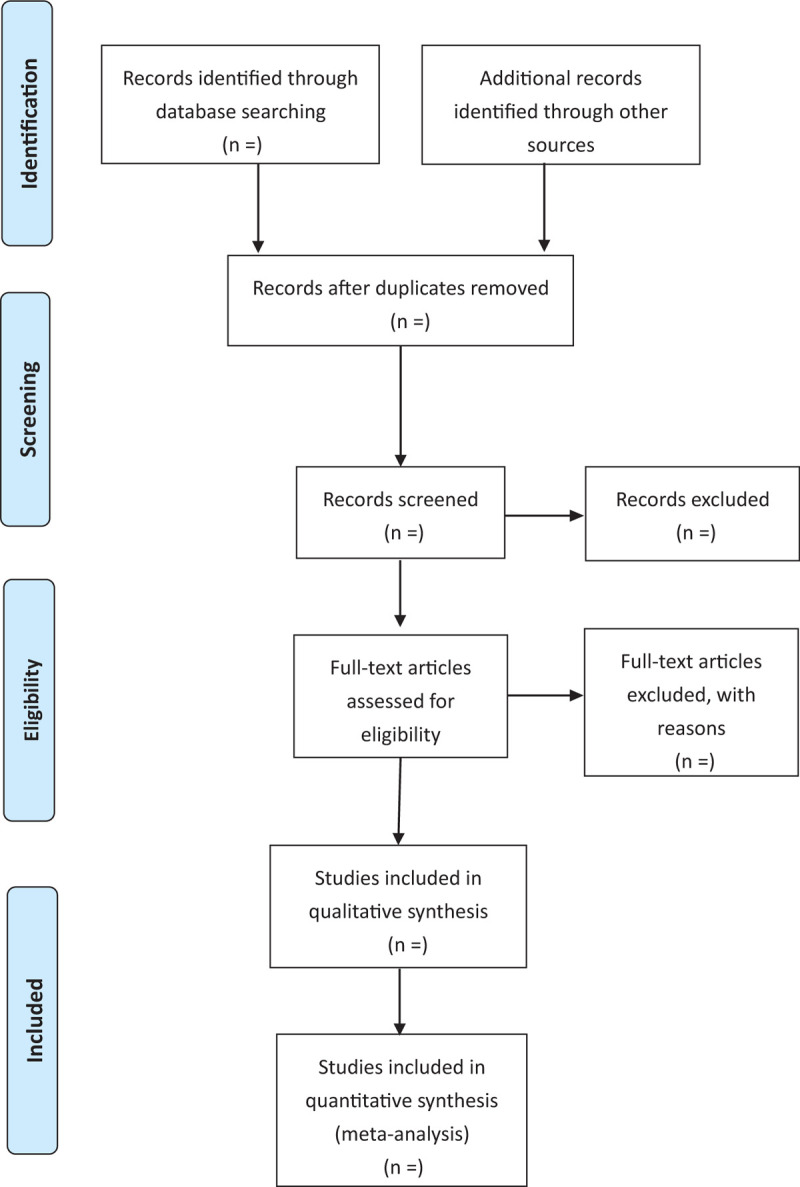
PRISMA flow diagram.

Literature screening and data extraction are independently completed by 2 researchers (MQ Liu and YY Li), such as preliminary screening, full-text reading, methodological analysis, and literature verification. All search results will be imported into EndNote and with build-in software, any duplicates will be removed. The full reports of potentially eligible articles are retrieved and inspected to assess their relevance against the inclusion criteria. Reference lists of relevant review papers are screened for potential studies. If there are any differences and disagreements in the questionable literatures, we will seek the opinion of the third reviewer (WL Yang). The general information form includes the basic information of the included literature, design and setting of the study, participants in the study, intervention measures, efficacy evaluation, outcomes, results, follow-up periods, and adverse events. The literature screening process is shown in Figure 1.

#### 2.4.2 Assessment and quality of included studies

To assess the quality of the included studies, 2 reviewers (MQ Liu and J Xian) independently assessed each study using the Cochrane Collaboration tool for systematic reviews.^[18]^ We will obtain the full text of all potentially relevant records. Divergence of views between the 2 reviewers is resolved by discussion and by consulting a third reviewer (WL Yang) when necessary. If we cannot resolve a disagreement, we will categorize the study as a “Study awaiting classification” and contact the study authors for clarification. The main items are:

(1) random sequence generation;
(2) allocation concealment;
(3) blind method for participants and implementers;
(4) blind method for outcome evaluation;
(5) incomplete outcome data;
(6) selective reporting;
(7) other bias.

The Cochrane risk assessment tool Rev man 5.3 is used to make the bias risk map and bias risk summary chart.

#### 2.4.3 Data extraction

The authors (MQ Liu and Q Gao) plan to extract the data from the articles selected for inclusion, including basic information about the studies, inclusion criteria, outcome measures, diagnostic criteria, inclusion completion, the demographics of the participants (age and gender), information on the participants, interventions, outcomes, follow-up results, presenting clinics, and the statistical analysis.

#### 2.4.4 Measures of treatment effect

The statistical analysis is conducted using Review Manager Software (Review Manager 5.3) by 2 authors (MQ Liu and YY Li). Continuous data are represented by standardized mean difference (SMD) and 95% confidence interval. The count data of categorical variables are expressed by odds ratio (OR) or relative risk.

#### 2.4.5 Dealing with missing data

There may be missing data in the literature, we will take the initiative to contact the corresponding author via email or other contact methods. If the missing data is not available, we will analyze the existing data that is assumed to be missing at random.

#### 2.4.6 Assessment of heterogeneity

The heterogeneity of the data will be assessed by *Q*-test and *I*^2^ statistic. The following criteria will be used: *I*^2^ < 50% will be considered as low heterogeneity; *I*^2^ between 50% and 75% will be deemed as moderate heterogeneity; *I*^2^ > 75% will be deemed as high heterogeneity.

#### 2.4.7 Assessment of reporting bias

We will use Revman to draw funnel plots to evaluate publication bias when there more than 8 studies be included. The symmetry of the funnel chart is affected by many factors, and publication bias is the main factor affecting the funnel chart.

#### 2.4.8 Data synthesis

The statistical analysis was performed using Cochrane Collaboration's Review Manager Software. RevMan software is able to conduct a meta-analysis and present the results graphically. A meta-analysis will be carried out based on measurement methods, intervention methods, heterogeneity levels, etc. If clinical and methodological heterogeneity are low, the fixed-effect model will be used for merger analysis; the random-effects model will be applied by merger analysis when heterogeneity indicates a moderate level. The overall quality of the evidence for the prespecified outcomes was assessed using the GRADE system,^[19]^ which including limitations of the research process, indirect evidence, low accuracy of results, inconsistent research results, and publication bias. The report of this systematic review and meta-analysis followed the PRISMA statement.^[20]^

#### 2.4.9 Subgroup analysis

According to the difference of intervention measures and curative effect indexes, the patients were divided into subgroups and then analyzed. For instance, if the heterogeneity is caused by particular features (eg, participants’ age, course of disease, treatment time, observation period, pediatric/adult massage, measurement methods), subgroup analysis will be conducted relevant to these categories.

## 3 Discussion

In summary, primary monosymptomatic nocturnal enuresis does not cause acute harm to children, but long-term nocturnal enuresis often brings greater disease burden and psychological pressure to children and their families, and seriously affects their quality of life and physical and mental growth. Once the diagnosis of nocturnal enuresis in children requires early treatment, clinicians and parents should not take a wait-and-see attitude. The large number of clinical practices further verified the safety and effectiveness of massage therapy. Pediatric tuina (massage) is a very good drug replacement therapy. It exerts the therapeutic action by performing the operation directly on the patient and using the simple physical principles, including pushing, holding, kneading, pressing, and other means of direct stimulation. The operation is simple, safe, and no side effects, through the use of hands directly on the body of patients. There have been more and more clinical reports on the treatment of primary MNE by pediatric tuina in recent years. There is still lack of reliable evidence for the efficacy and high-quality trials. This study aims to organize and summarize the relevant literature on the treatment of primary MNE by pediatric tuina. The results of this trial will help doctors have a deeper understanding of Tuina therapy and will provide valuable evidence for future research in this area. To provide convincing evidence and better guidance for clinical practice, all actions of this review will be performed according to Cochrane Handbook 6.0.

## Author contributions

**Conceptualization:** Muqing Liu, Juan Yu.

**Data curation:** Muqing Liu, Yingying Li, Wenlong Yang.

**Formal analysis:** Juan Yu.

**Investigation:** Muqing Liu, Jin Xian, Qing Gao.

**Methodology:** Muqing Liu, Yingying Li.

**Supervision:** Muqing Liu, Wenlong Yang.

**Validation:** Juan Yu, Jin Xian.

**Visualization:** Muqing Liu.

**Writing – original draft:** Muqing Liu.

**Writing – review & editing:** Muqing Liu, Juan Yu.
